# The effect of direct and extended contact on attitudes towards social robots

**DOI:** 10.1016/j.heliyon.2021.e06418

**Published:** 2021-03-27

**Authors:** Marina Sarda Gou, Thomas L. Webb, Tony Prescott

**Affiliations:** a Department of Psychology, The University of Sheffield, UK; b Department of Computer Science, The University of Sheffield, UK

**Keywords:** Human-robot interaction, Contact theory, Extended contact, Attitudes, Social robotics

## Abstract

The development of social robots has the potential to address significant societal concerns, however, most people have limited experience of such technology. The present research investigated whether techniques borrowed from the psychology of intergroup relations – namely direct and extended contact – affect people's attitudes towards robots. Participants were provided with either direct contact with a social robot or extended contact (these participants watched a video recorded by a friend who had met the robot) before their explicit and implicit attitudes towards robots were measured. Results indicated that direct contact affected both explicit and implicit attitudes, while extended contact affected implicit attitudes. The implication of these findings is that contact with a robot, direct or indirect, can change attitudes; much as previous research has shown that contact with a person who is a member of an out-group can change attitudes towards that group. We conclude that methods and theories from the study of human intergroup relationships can be usefully applied to understand attitudes toward social robots.

## 1 Introduction

Rapid advances in technology mean that it is possible to develop ‘social’ robots to assist people in their day-to-day lives. A social robot is an embodied system that can be perceived of as a social entity and that is capable of communicating with the user (Broekens et al., 2009). While many social robots have been developed with older people and the disabled in mind (Bogue, 2013), social robots may be useful and relevant for all members of society (Dario et al., 2011; Prescott, 2017), with robots being developed for companionship (Odetti et al., 2007) and assistance in public places working in areas such as retail, tourism, hospitality, and so on (Hans et al., 2002; Harmo et al., 2005; Ivanov et al., 2017; Kachouie et al., 2014; Yamazaki et al., 2007). However, people are often wary of new technologies such as robots and Artificial Intelligence (AI); an unease that can be reinforced by public figures and media. Indeed, AI is typically illustrated in the popular press using images of threatening fictional robots such as those from the “Terminator” film series. Published research on people's attitudes towards robots corroborates the existence of this unease. For example, there is an established literature on robot anxiety (Nomura et al., 2008) and evidence that people often associate robots with weapons (MacDorman et al., 2009).

Nervousness about the prospect of social robots is perhaps not surprising – people have limited contact with such technologies and so it is difficult for the wider public to understand how robots function (Kriz et al., 2010). Indeed, as noted above, attitudes and beliefs are often based on media representations, science fiction literature and films (Kriz et al., 2010), rather than on real-world examples. It therefore seems likely that providing people with the opportunity to find out about the contemporary reality of such technologies could influence their opinions.

The present research takes a novel approach to understanding attitudes towards robots, grounded in the psychology of intergroup relations. This research suggests that direct contact with members of stigmatized groups can challenge negative preconceptions and reduce prejudice. In his book on the nature of prejudice, Gordon Allport introduced the “contact hypothesis” which states that, under the right conditions, contact between members of different groups can improve intergroup relations and lessen hostility (Allport, 1954). The present research combines this idea with Reeves and Nass' (1996) “media equation”— which states that people often treat computers and other new media as if they were human beings – to propose that the contact hypothesis might be applied to people's relations with artificial agents such as robots.

There is some evidence that direct contact with robots can influence people's beliefs (Nomura et al., 2008; Nomura et al., 2011). However, previous research examining the effect of direct contact with robots on attitudes has relied on self-report (and thus explicit) measures of attitudes. Explicit attitudes are consciously available to introspection (Hahn et al., 2014), that is to say, people can think about them. Given that sometimes people are not aware of the attitudes that affect their behaviour (MacDorman et al., 2009), or may be tempted to respond in a way that they believe to be socially desirable or expected (cf., demand effects), it is not surprising that there are instances when explicit and implicit attitudes may diverge (Wilson et al., 2000). Therefore, it is important to extend studies examining the effect of direct contact with robots to examine the effects of direct contact on *implicit* attitudes. Implicit attitudes are activated automatically without the person's awareness (Greenwald and Banaji, 1995) and so may be less influenced by self-presentational concerns or desirable responding.

Furthermore, direct contact may not always be possible, especially in the case of novel technologies like social robotics. In an effort to address the lack of opportunities for contact, psychologists interested in intergroup relations have investigated the effects of indirect forms of contact. One form of indirect contact is extended contact, which involves learning that an in-group member is friends with an out-group member (Dovidio et al., 2011; Zhou et al., 2019). Wright et al. (1997) claimed that “*an in-group member engaged in a close friendship with a member of the out-group should provide a salient and effective source of referent informational influence, demonstrating positive intergroup attitudes and tolerant in-group norms*” (Wright et al., 1997, p. 75). In support of this idea, Wright et al. found that participants who knew that an in-group member was friends with someone in the target out-group showed significantly less prejudice towards that out-group. Translating these findings to the field of human-robot interaction, extended contact could also provide people with an opportunity to learn about robots and influence their attitudes towards robots.

## 2 The present research

The aim of the present research was to examine the effects of direct and extended contact on people's implicit and explicit attitudes toward social robots. Our hypothesis was that attitude formation and change with respect to social robots would show similar dynamics to attitude change with respect to interpersonal relations; particularly, with respect to people's attitudes toward members of minority groups with whom they rarely have contact. In other words, based on our extension of Allport's contact hypothesis to human-robot interaction, we predicted that both direct and extended contact with social robots would influence attitudes towards this technology.

Two studies were carried out to test this hypothesis, the second as a preregistered partial replication of the first, intended to test the robustness of the findings relating to effects of extended contact on attitudes. The main difference between the first study and its replication is the number of conditions that they had. The first study had four conditions (direct contact, extended contact, no contact control, and extended contact control) while the replication study only had two (extended contact and no contract control) as the aim of the second study was simply provide a second test of the effect of extended contact on attitudes. In addition, different robots were used in the two studies.

## 3 Study 1

### 3.1 Design

**Figure 1:**
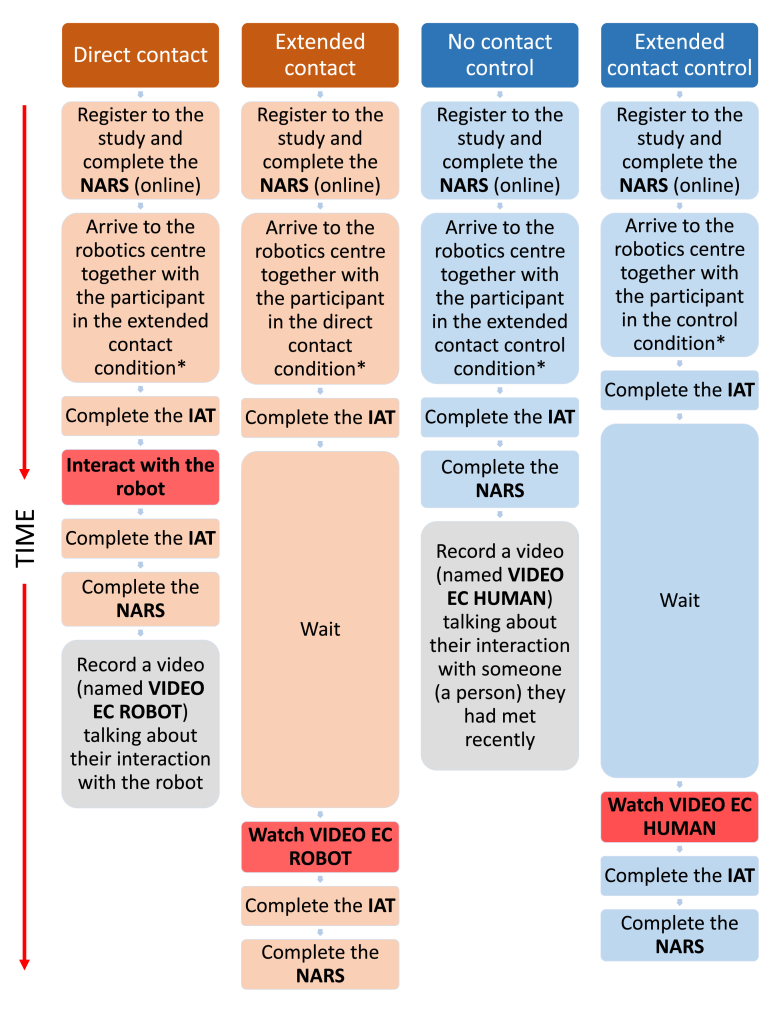
Experimental protocol for Study 1. Participants in the direct contact and extended contact conditions came together (orange) and they were in an experimental condition, in which an effect of the intervention was expected. Participants in the control and extended contact control conditions came together (blue) and no effect was expected since they were in control conditions. Actions marked in red represent the experimental interventions that could have affected participants' attitudes. The actions marked in grey represent procedures that did not affect the results because they were performed after all data was collected. ∗Participants did not know in which condition they were before taking part in the experiment.

Our first study adopted an experimental design with two experimental and two control conditions, as illustrated in Figure 1. This study used a mixed design including time as a within-participants factor (before and after contact), and contact as a between-participant factor (direct contact, extended contact, no contact control, and extended contact control). A power analysis was performed to estimate the required size of the sample based on a medium-sized (*d* = 0.48 or *f*^2^ = 0.24) effect of contact on attitudes according to Cohen's criteria (1988). With alpha = .05 and power = 0.95, GPower 3.1 estimated the sample size needed to detect an effect of this magnitude in a mixed ANOVA, to be *N* = 80 (40 pairs, with 20 pairs per condition).

Participants were students and staff from a large University in the North of England. They were recruited using either an online research participation system (the SONA system in the Department of Psychology at the University of Sheffield) or via an email distribution list containing staff and students who had indicated a willingness to take part in research. Participants who responded via either route were asked to identify a friend, or someone who was closer than a friend, who could also participate in the study. Both participants had to be aged over 18 and consent to taking part in the research. Participants who were recruited via the online system (SONA) received 4 course credits for their participation, all other participants received no incentive.

### 3.2 Demographics

80 participants, that is 40 friendship pairs, took part. The participants average age was 23.86 (*SD* = 8.03); 30 were male and 50 were female and the majority were British (*n* = 62, 78%). There is no conclusive empirical evidence indicating a gender difference in attitudes towards robots (Naneva et al., 2020) so the gender of participants was not considered further. The mean score on the Inclusion of Others Scale (Aron et al., 1992) was 4.97 (*SD* = 1.55), which indicates that participants knew each other at least reasonably well. Out of 40 pairs of participants, 27 pairs were friends, 11 pairs were dating exclusively or married, and 2 pairs were mother and daughter.

Participants previous experience with robots might influence their attitudes toward robots (Kachouie et al., 2014; Leite et al., 2013; Syrdal et al., 2014). Therefore, participants were asked four questions. The first question was “How many films or TV shows have you seen in which there are humanoid robots?” Participants could select one of 5 answers which went from 0-5 films (*N* = 44, 55%), 6–10 films (*N* = 22, 28%), 11–15 films (*N* = 3, 4%), 16–20 films (*N* = 7, 9%) or 20 + films (*N* = 4, 5%). The second question was “Have you ever seen a humanoid robot in real life?”. Participants could answer yes (*N* = 15, 19%) or no (*N* = 65, 81%). The third question was “Have you ever interacted with a humanoid robot?” (Yes, *N* = 3, 4%; No, *N* = 77, 96%). Finally, we also asked participants if they had ever controlled a humanoid robot. None of the participants reported that they had. Because only a small percentage of participants had some previous experience with humanoid robots, it was not possible to examine the effects of this factor.

### 3.3 Measures

Explicit attitudes were measured using the *Negative Attitudes towards Robots Scale* (NARS) (Nomura et al., 2006) which has been used extensively in the field of Human-Robot Interaction (HRI) (Naneva et al., 2020; Syrdal et al., 2009). The NARS has 14 items (e.g., *I would feel uneasy if robots really had emotions*) to which participants respond on a Likert scale from 1 to 5 where 1 corresponds to the most positive attitudes towards robots and 5 the most negative attitudes. Participants' responses were computed to create a single score reflecting their explicit attitudes towards robots.

**Figure 2:**
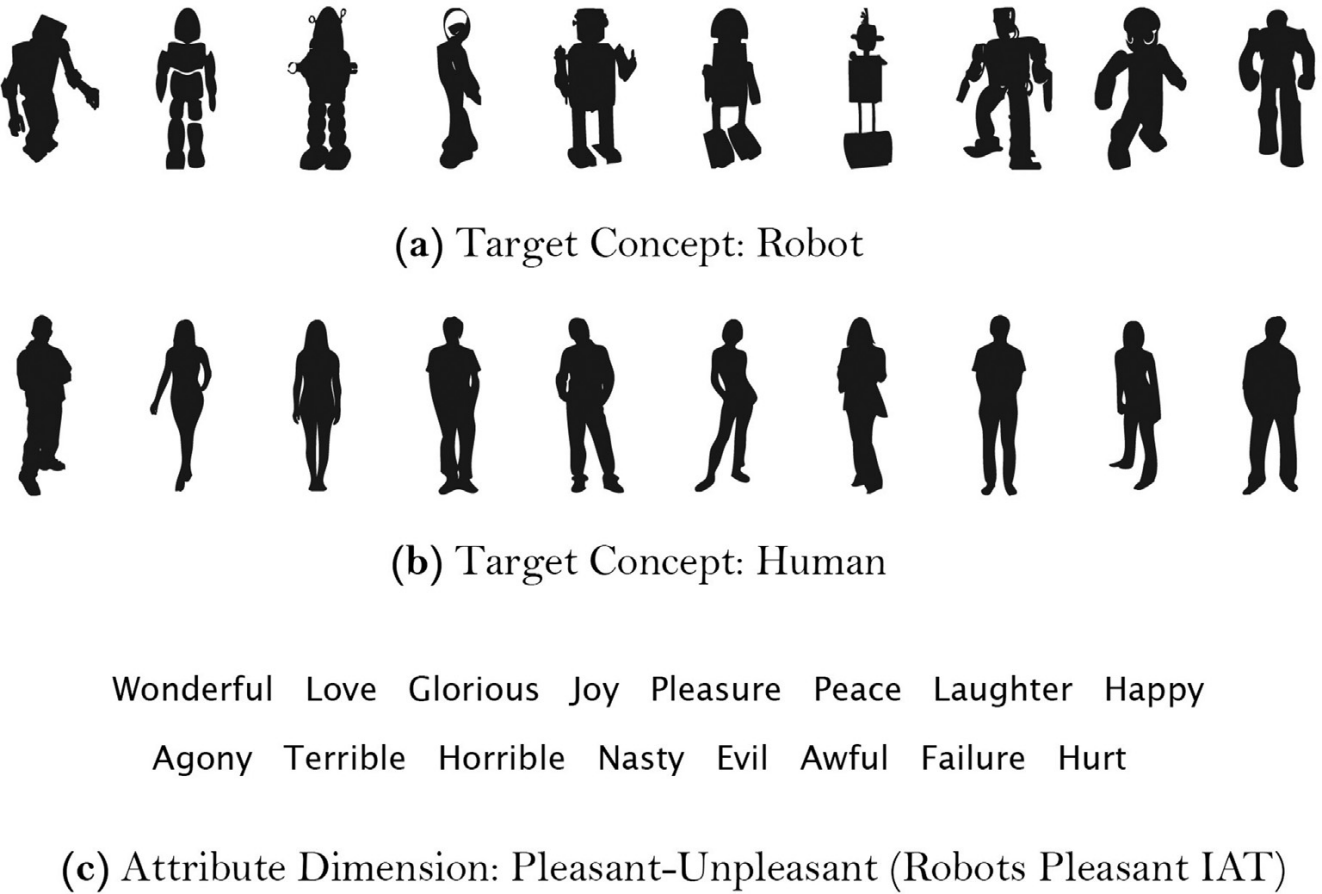
Images and words used by MacDorman et al. (2009) in the IAT. Reprinted with permission from Springer Nature Customer Service Centre GmbH.

Implicit attitudes were assessed using an adapted version of the *Implicit Attitude Test* (IAT; Greenwald et al., 1998) designed to measure implicit attitudes towards robots (MacDorman et al., 2009). It uses ten silhouettes of humans and ten silhouettes of robots as targets, and eight pleasant words and eight unpleasant words as attributes (see Figure 2). The IAT asks participants to sort stimuli, such as pictures or words, into groups. The test works according to the well-established finding that participants are quicker to sort stimuli about which they hold similar views if those stimuli share a response key (Greenwald et al., 1998). For example, if a participant has a favourable view of robots, then they should respond more quickly when “robot” and “good” categories are combined (e.g., Press key “A” if a robot or word reflecting something good appears) than when “robot” and “bad” categories are combined (e.g., Press key “A” if a robot or word reflecting something bad appears). In order to obtain a value that represented participants' implicit attitudes, a D score was calculated using the algorithm described by Greenwald et al. (2003); higher D scores reflect more negative implicit attitudes towards robots.

The *Inclusion of Other in the Self* scale (IOS) (Aron et al., 1992) was used to measure how close a participant felt to their friend. In addition, they were asked to write how they had met each other. Participants were in separate rooms while completing this questionnaire and could not see what their friend had written in the questionnaire. Responses to the IOS were highly congruent between pairs (Pearson's *r* = .85).

### 3.4 Robot interaction

Participants interacted with the *Softbank Pepper* humanoid robot, as Pepper represents an example of a social humanoid robot. Participants in the direct contact condition watched an instructional video (see Video 1 or https://youtu.be/lDKT2FGN3ak) that asked them to pretend that they were in a shop and that Pepper was assisting them. The robot was programmed to have an interaction with the participant about hair products, using the software *Choregraphe*. A range of shampoo bottles was displayed on a shelf that the participant was invited touch and ask questions about. Initially, the robot introduced itself and thanked the participant for coming. The robot then proceeded to talk about the different hair care products, asking the participant about their hair type and recommending different shampoos. The participant could also ask questions about a specific shampoo. This interaction was limited to maximum of 5 min.

Supplementary video related to this article can be found at https://doi.org/10.1016/j.heliyon.2021.e06418

The following is the supplementary data related to this article:

Video 1 1 Video 1

### 3.5 Procedure

**Figure 3:**
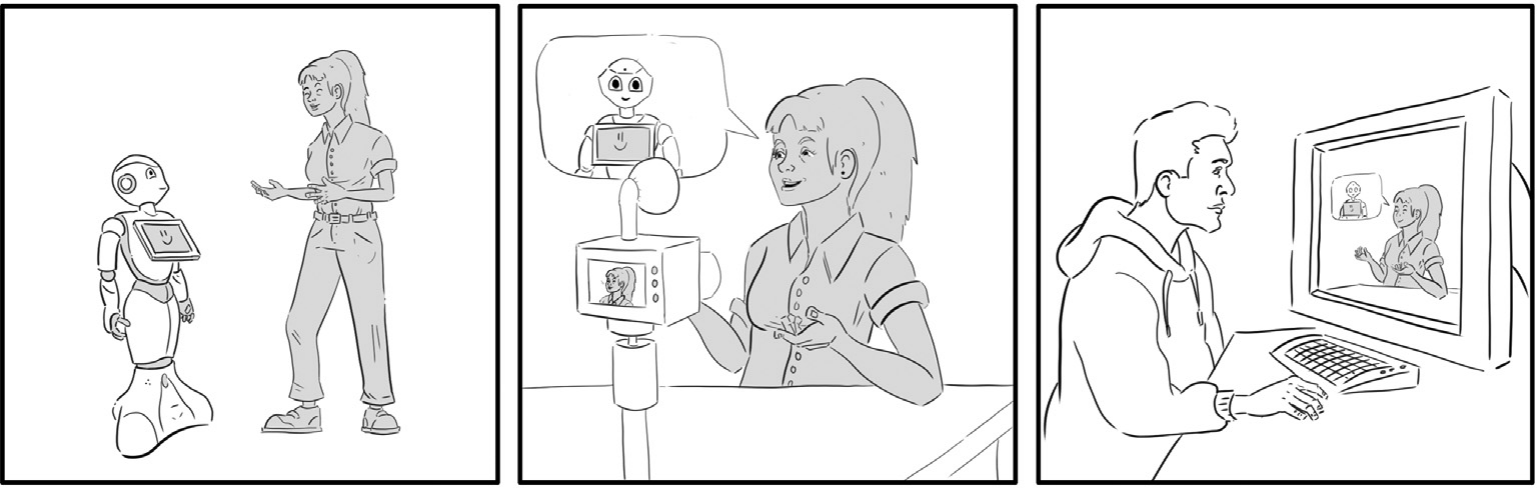
Experimental procedure. The first two vignettes show the participant in the DC condition in which they first interact with the robot and then record a video talking about the robot. In the third vignette, the participant in the EC condition watches the video that their friend has recorded. Illustration by Paula Garcia Gou.

**Figure 4:**
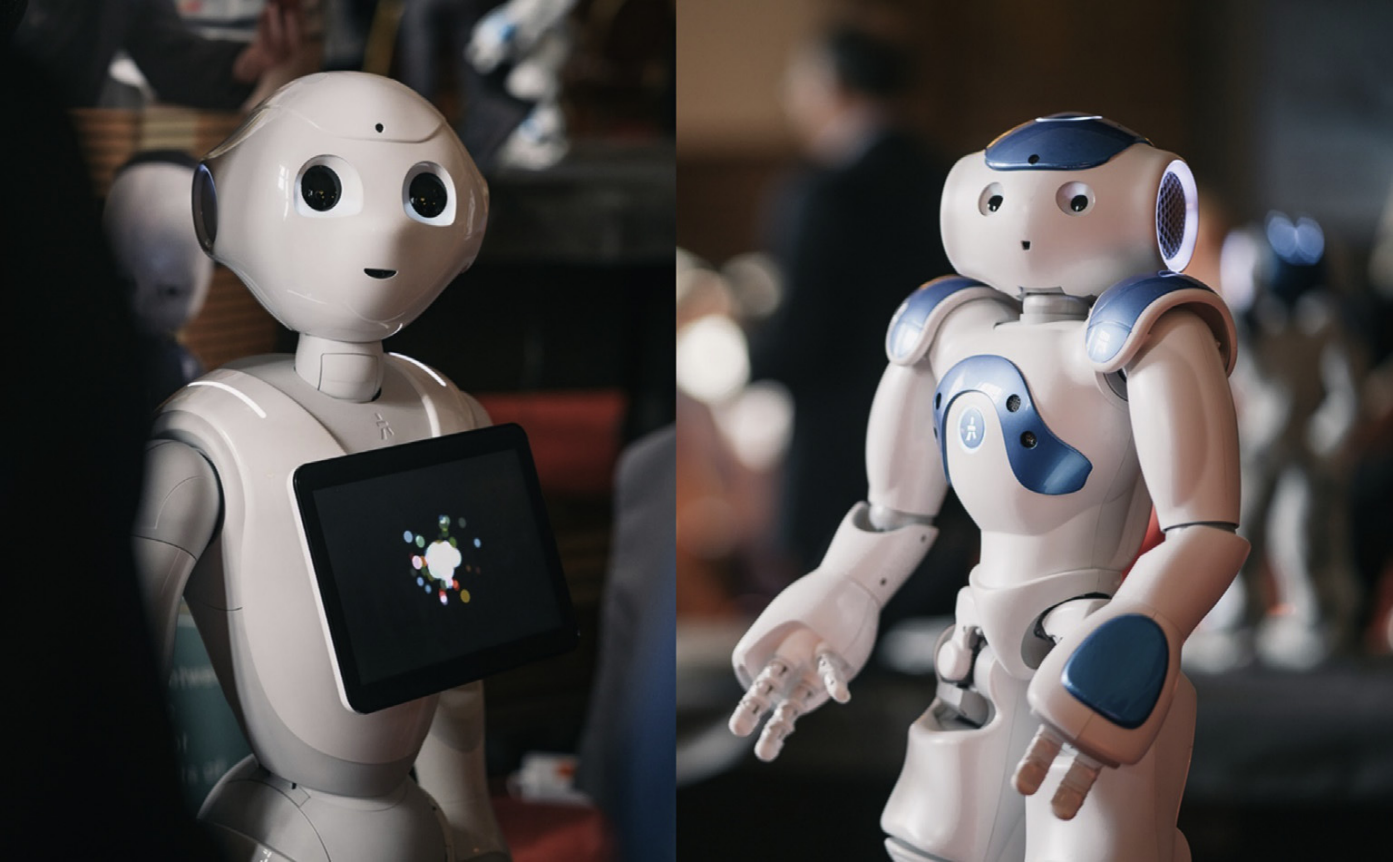
Robots used in the studies. Left, the Softbank Pepper robot, height 120cm, used in Study 1. Right, the Softbank Nao robot, height 57cm, used in Study 2. Photographs from the University of Sheffield.

Figure 1 illustrates the experimental protocol for Study 1. All participants (respondents and their friends) registered online and completed a demographic survey and the NARS questionnaire. When the two participants arrived at the robotics centre, they separated into two different rooms and asked to complete the IAT (to measure their implicit attitudes) and the IOR. They were then randomly allocated to either an experimental or control condition and the procedure that followed then differed between conditions:

Participants in the direct contact condition (Figure 3, first and second vignette) interacted for five minutes with *Pepper* (Figure 4, left), in a setting in which the human and robot engaged in a conversation about hair products. After the interaction, the participant completed the NARS and IAT a second time before recording a short video message for their friend describing their experience with the robot. Specifically, each participant was asked to answer the following questions while talking to the camera:

*What happened since you first saw Pepper until the end of the interaction?*

*What did you talk about?*

*How did you feel while interacting with Pepper?*

*Did Pepper help you to achieve the purpose of the conversation (i.e., choosing the product)?*

*Did you like Pepper? Why?*

Most participants seemed to like Pepper and were happy with the recommendations that Pepper provided. For example, participants said: “*We talked about shampoos. It was delightful.*”, “*He made it feel more comfortable as it was easy to talk to and clearly understood what I was saying*”, and “*I was quite impressed with Pepper's dialect and the sort of flow with conversation.*” After recording the video, the participant then left the room and their friend, who was in the extended contact condition (Figure 3, third vignette), came in and watched the recorded video, they also completed the NARS and IAT measures a second time.

There were two control conditions. Participants in the no contact control condition also had already completed the NARS online and the IAT once in the lab (like any other participant). They did not complete a second IAT (since no interaction took place) but they completed the NARS a second time once they were in the lab without interacting with anything or watching any video. After that, they were instructed to record a short video talking about someone (human) that they had met recently for the first time (e.g., a new friend, a shop assistant, a waiter, a taxi driver, a receptionist). Specifically, they were asked to answer the following questions while talking to the camera:

*What happened with this person since you began this conversation until you finished talking?*

*What did you talk about?*

*How did you feel interacting with this person?*

*If the conversation had a purpose did this person help you to achieve the purpose of the conversation?*

*Did you like this person? Why?*

The participant then left the room and their friend, who was in the extended contact control condition, came in and watched the recorded video, before completing the NARS and IAT measures a second time. The extended contact control condition therefore served to separate the effects of extended contact (e.g., with another human being) from the specific effects of extended contact with a robot, as reflected by the extended contact condition.

It is worth noting that recording a video talking to the camera was not intended to serve as an intervention in the direct contact and the no contact control conditions – indeed, the video was recorded after participants had already completed all the measurements and all the data was collected. Therefore, it could be said that they recorded the video *after* taking part in the study. The sole purpose of asking these participants to record a video describing their experiences was to generate realistic and ecologically valid videos that could be used to examine the effects of extended contact with a robot (the extended contact condition), controlling for extended contact more generally (the extended contact control condition).

At the end of the study, all participants were debriefed and had the opportunity to ask questions.

### 3.6 Results

To examine the effects of direct and extended contact on explicit attitudes, we conducted a 4-between (condition: direct contact, extended contact, no contact control, extended contact control) by 2-within (time: before vs. after) mixed ANOVA with NARS scores as the dependent variable (see Table 1). There was a significant effect of time on explicit attitudes, *F*(1, 76) = 7.15, *p* = .009, partial eta^2^ = .09, that was qualified by a significant interaction between condition and time, *F*(3, 76) = 3.56, *p* = .018, partial eta^2^ = .12. Follow-on paired sample t-tests indicated that explicit attitudes changed as a function of direct contact, *t*(19) = 2.86, *p* = .010; *d* = .64; such that participants had more positive explicit attitudes towards robots after interacting with *Pepper*, but that explicit attitudes did not change significantly as a result of extended contact, *t*(19) = 0.24, *p* = .817, or either of the control procedures: extended contact control condition, *t*(19) = 1.07, *p* = .300, and control condition, *t*(19) = 0.06, *p* = .949.

**Table 1:** Implicit and explicit attitudes by time and condition (study 1).

	Direct contact				Extended contact			
	Explicit attitudes*		Implicit attitudes*		Explicit attitudes		Implicit attitudes*	
	Before	After	Before	After	Before	After	Before	After
Mean	2.82	2.48	0.50	0.31	2.75	2.74	0.41	0.25
*SD*	0.70	0.51	0.35	0.38	0.66	0.63	0.36	0.32
	Extended contact control				Control			
	Explicit attitudes*		Implicit attitudes*		Explicit attitudes		Implicit attitudes*	
	Before	After	Before	After	Before	After	Before	After

Mean	2.72	2.64	0.44	0.34	2.80	2.79	0.54	0.54
*SD*	0.53	0.62	0.36	0.30	0.53	0.57	0.30	0.30

*Note*. * indicates a significant (*p* < .05) difference between before and after assessments.

To examine the effect of condition on implicit attitudes we conducted two 4-between ANOVAs with the before and after implicit attitude scores in each condition compared with the single implicit attitude score in the no contact control condition. There was no significant differences between the before contact scores, *F*(3, 76) = .63, *p* = .598, partial eta^2^ = .02, but there was a significant main effect, *F*(3, 76) = 3.00, *p* = .036, partial eta^2^ = .11 for after contact scores, indicating that participants in the extended contact condition had more positive implicit attitudes (*M* = .25, *SD* = .32) than those in the no contact control condition (*M* = .54, *SD* = .30, Tukey, *p* = .046). Follow-up paired sample t-tests indicated that implicit attitudes became more positive as the result of both direct contact, *t*(19) = 3.05, *p* = .007; *d* = .68, and extended contact, *t*(19) = 2.49, *p* = .022; *d* = .56. Implicit attitudes did not change significantly in the extended contact control condition, *t*(19) = .93, *p* = .364, *d* = .29.

### 3.7 Discussion

The findings of Study 1 were largely consistent with the hypothesis that the Contact Hypothesis (Allport, 1954) can be extended to understand how contact with non-human agents (e.g., social robotics) influences people's attitudes. In particular, we found that direct contact with a social robot had a positive effect on participants' explicit and implicit attitudes, while extended contact only had a positive impact on implicit attitudes. Since the effects of extended contact were only partially as expected, we conducted a second study replicating the extended contact condition in Study 1 but with a different robot and interaction setting.

## 4 Study 2: conceptual replication

### 4.1 Design

Study 2 partially replicated the design of Study 1, using the *Softbank Nao* humanoid robot in place of *Pepper* and in a setting where the robot recommended films to participants. Nao was chosen because it is different in size and shape but otherwise similar to Pepper (e.g., Nao also has a humanoid face and voice). Since both studies are focused on humanoid robots, there was a need to have two robots that matched this characteristic while being different enough in order to reduce the possibility that the findings are specific to a particular robot. Study 2 examined only the effect of extended contact on attitudes, compared to no contact. The procedures and approach to analysis were pre-registered on AsPredicted.org (#17464).

Study 2 used a mixed design including time as a within-participants factor (before and after extended contact), and two between-participant factors—no contact and extended contact. A power analysis was performed to estimate the required size of the sample based on a medium-sized difference between extended contact and control conditions (*d* = 0.48, which equates to effect size *f*^2^ = 0.24) as we did in the previous study, but with a lower power threshold (0.80) since study 1 gave us more confidence of an effect. With alpha = .05, power = 0.80, and two conditions, GPower 3.1 recommended a sample size of *N* = 38, or 19 pairs.

### 4.2 Demographics

46 participants, or 23 friendship pairs, took part. Their mean age was 23.24 (*SD* = 10.14); 15 of them were male and 31 were female; the majority were British (*N* = 36, 78%). Up until now, there is no conclusive empirical evidence indicating a gender difference in attitudes towards robots (Naneva et al., 2020) The average IOS for all participants was 4.89 (*SD* = 1.50), which indicates that participants knew each other. Out of the 23 pairs of participants, 18 pairs were friends, 4 pairs were dating exclusively or married, and 1 pair were mother and daughter. Responses to the IOS were highly congruent between pairs (Pearson's r = .70).

As in the previous study, participants were also asked about their previous experience with humanoid robots. The first question was “How many films or TV shows have you seen in which there are humanoid robots?” Participants could select one of 5 answers which went from 0-5 films (*N* = 26, 57%), 6–10 films (*N* = 13, 28%), 11–15 films (*N* = 2, 4%), 16–20 films (*N* = 0, 0%) or +20 films (*N* = 5, 11%). The next question was “Have you ever seen a humanoid robot in real life?” (Yes, *N* = 5, 11%; No, *N* = 41, 89%). The following one was “Have you ever interacted with a humanoid robot?” (Yes, *N* = 1, 2%; No, *N* = 45, 98%). Finally, we also asked participants if they had ever controlled a humanoid robot and all of them reported that they never had. Because only a small percentage of participants had some previous experience with humanoid robots, it was not possible to examine the effects of this factor in the present study.

### 4.3 Procedure

**Figure 5:**
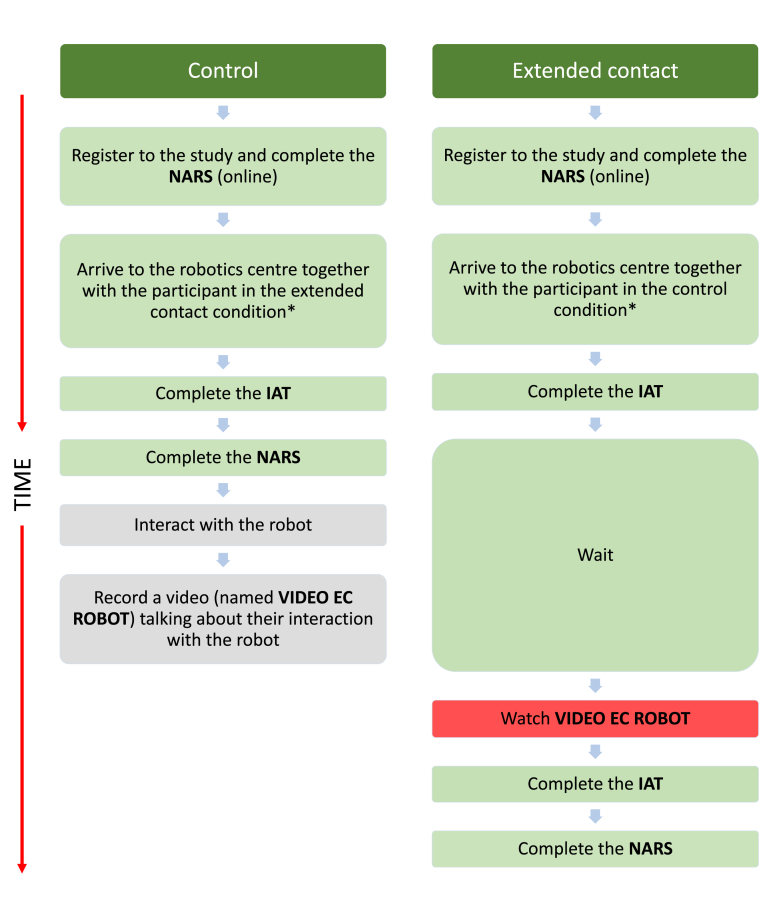
Experimental protocol for Study 2. Participants in the control and extended contact conditions came together. The action marked in red represent the experimental intervention that could have affected participants' attitudes. The actions marked in grey represent procedures that did not affect the results because they were performed after all data was collected. ∗Participants did not know in which condition they were before taking part in the experiment.

Figure 5 illustrates the experimental protocol for Study 2. Participants were recruited in pairs again, in the same way as in Study 1, and the same measures were used in order to assess participants' relationship to each other (IOR) and attitudes towards robots (NARS and IAT). They completed measures of explicit attitudes towards robots online before visiting the lab, and measures of implicit attitudes on arriving at the lab. Once in the lab, the members of each participant pair were randomly allocated either to the no contact condition or to the extended contact condition and asked to wait in separate rooms.

Participants in the no contact condition were first asked to complete the NARS a second time and the IAT. After they had completed all the measures, they watched an instructional video (available as Video 2 or https://youtu.be/YRxN2w2WMak), and films were placed on a table in front of the participant. These participants then interacted with the *Softbank Nao* humanoid robot (Figure 4, right). At the beginning of the interaction, the *Nao* robot introduced itself and thanked the participant for coming. The robot then talked about the films, asked the participant about their taste in films, and provided some recommendations. Participants could also ask the robot questions about the films. The interaction was limited to 5 min.

Supplementary video related to this article can be found at https://doi.org/10.1016/j.heliyon.2021.e06418

The following is the supplementary data related to this article:

Video 2 2 Video 2

After that, participants recorded a short video describing their interaction with the robot, the questions were the same as in Study 1, replacing “Pepper” with “Nao.” Most participants said that they liked Nao and were happy with the recommendations that Nao provided. For example, participants' said: “It felt very like real, very like a proper human interaction.”, “Nao was the first robot I interacted with. So, it was an amazing experience actually. It was fun and it was very… I learnt a lot about Nao”, and “He is a friendly little fellow and it moves around. It is quite like lifelike. I like that.”

It is worth noting that this contact with the robot did not affect these participants' measures as they interacted with the robot after all data was collected. Therefore, it could be said that they had contact with the robot and recorded a video talking about their interaction after their participation in the study. The sole purpose of this interaction was to record the video that then would be used in the extended contact condition.

The participant then left the room and their friend, who was in the extended contact condition, came in and watched the recorded video, before completing the NARS and IAT attitudes measures a second time.

### 4.4 Results

A 2-between (condition: no contact vs. extended contact) by 2-within (time: before vs. after) mixed ANOVA with NARS scores as the dependent variable was used to test if there was any change in explicit attitudes as a function of extended contact (see Table 2). There were no significant effects of condition, *F*(1, 44) = 1.17, *p* = .285, partial eta^2^ = .03, time, *F*(1, 44) = 1.24, *p* = .272, partial eta^2^ = .03, or the interaction between condition and time, *F*(1, 44) = 0.29, *p* = .592, partial eta^2^ = .01, on explicit attitudes as measured by the NARS.

**Table 2:** Implicit and explicit attitudes by time and condition (study 2).

	Control				Extended contact			
	Explicit attitudes		Implicit attitudes		Explicit attitudes		Implicit attitudes∗	
	Before	After	Before	After	Before	After	Before	After
Mean	2.65	2.57	0.55		2.79	2.76	0.56	0.38
*SD*	0.59	0.60	0.23		0.48	0.42	0.25	0.34

*Note*. ∗ indicates a significant (*p* < .05) difference between before and after assessments.

With respect to implicit attitudes, there was no significant difference between the conditions before the extended contact procedure, *t*(44) = -0.10, *p* = .921. There was however, a significant effect of time on implicit attitudes in the extended contact condition, *t*(22) = 2.45, *p* = .023; *d* = .51; indicating that, as in Study 1, indirect contact with the robot led participants to hold more positive implicit attitudes towards robots.

### 4.5 Discussion

Study 2 replicated the effects of extended contact on attitudes toward robots as identified in Study 1. Taken together these results suggest that extended contact with a robot affects implicit, but not explicit attitudes toward robots.

**Figure 6:**
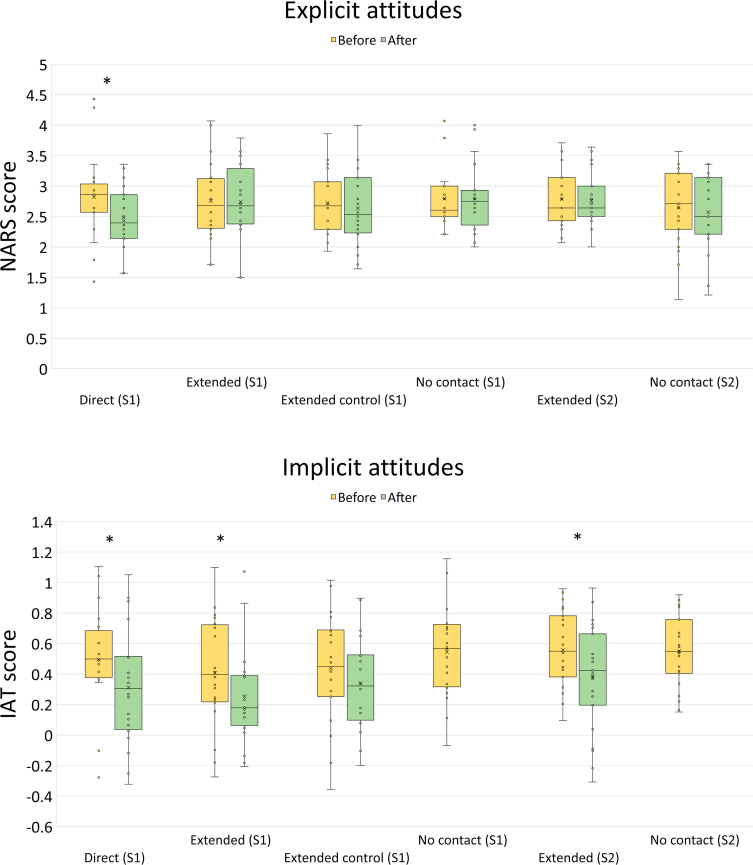
Results. Effect of different forms of contact and related controls, in studies 1 (S1) and 2 (S2), on measures of explicit and implicit attitudes (∗p < 0.05). A positive change in attitudes corresponds to a reduced score on either scale. A positive effect of contact on explicit attitudes was found only for direct contact, however, implicit attitudes were more positive following both direct and extended contact in both studies. Control conditions did not induce any significant changes in attitudes. See the Supplementary Material for a table of means and standard deviations.

Figure 6 shows the effects of direct and extended contact on participants' explicit and implicit attitudes towards robots across both studies. Note that a positive change in attitude is indicated by a *lower score* on both the explicit and implicit measures (i.e., less negative attitudes). In both studies, the effect sizes for the key comparisons are between partial eta^2^ = 0.09 and 0.25 indicating medium-sized effects. Cohen's *d* for the significant paired sample comparisons all exceeded 0.5 also indicating medium-sized effects (Cohen, 1988).

## 5 Discussion and conclusion

The present research drew on the psychology of intergroup relations to investigate the effects of social contact (direct and extended) on attitudes toward ‘social’ technologies; in this case, social robots. The findings suggested that direct contact with a social robot had a positive effect on both explicit and implicit attitudes towards robots. Taken together with previous studies showing positive effects of direct contact with robots on explicit attitudes (Nomura et al., 2008, 2011), these findings strengthen the conclusion that Allport's (1954) contact hypothesis can be extended to non-human agents. While beneficial, however, direct contact is often not possible, especially with advanced technologies such as social robots. Therefore, we also examined the effect of extended contact, in which the participant heard about a friend's interaction with a social robot. The findings suggested that participants had more positive implicit, but not explicit, attitudes towards robots after watching a video of a friend describing their interaction with a robot. This again supports the application of the contact hypothesis to relations between humans and social robots.

Contrary to our initial expectations, however, we found no evidence, in either study, that extended contact influenced participants' *explicit* attitudes. Previous research suggests that discordance between implicit and explicit measures is common (Echabe, 2013; Gawronski et al., 2020). For example, some studies have found that explicit attitudes can be changed more easily than implicit attitudes (Gawronski and Strack, 2004; Gregg et al., 2006; Petty et al., 2006), while others have found the opposite (Barden et al., 2004; Dasgupta and Greenwald, 2001; Wittenbrink et al., 2001). One possible explanation for the absence of an effect of extended contact on explicit attitudes is that participants may not have been aware of the effect that watching the video had on their attitudes – in other words, extended contact served as a ‘supraliminal’ priming procedure (Bargh and Chartrand, 2000). Indeed, previous research has shown that implicit attitudes can be shaped by recent experiences (Barden et al., 2004; Lowery et al., 2001; Mitchell et al., 2003) and that these changes may occur outside of explicit awareness or conscious control.

Alternatively, participants may have interpreted extended contact (but not direct contact) as intended to change their opinion and resisted this potential influence. A meta-analysis of the effects of selective exposure to information (Hart et al., 2009) suggested that, in some cases, people do not change their opinion even if they have evidence that challenges their beliefs. It is therefore possible that participants did not want to change their attitudes only by hearing a friend's opinion, or that this was experienced as a more overt attempt to change attitudes compared to direct interaction with the robot. Future research could probe participants' awareness of the aims of the contact manipulations to distinguish between these alternatives.

### 5.1 Implications

From a practical perspective, our findings suggest that direct and extended contact might be used as to provide people with experience of social robots and ground attitudes closer to reality, rather than, for example, science fiction. Contact provides knowledge about the outgroup and reduces anxiety about intergroup contact (Pettigrew and Tropp, 2008). Furthermore, extended contact requires minimal or no equipment, is affordable, and can be done in many contexts and settings. For example, Cameron et al. (2006) read stories to children about friendships with out-group members, which led the children to have more positive attitudes toward refugees. Similar procedures might be used to ground children's attitudes toward robots in reality. Theatre has also been used as a tool for measuring participants' views of human-robot interaction (Chatley et al., 2010; Walters et al., 2013), and the present findings suggest that such experiences might constitute a form of extended contact and thus serve to shape participants' beliefs.

The present research also has implications for those working in the field of HRI, as it provides further evidence that techniques developed in the social sciences (e.g., measures of attitudes, contact procedures) can be used to study the effects of interacting with robots or indeed, simply hearing about such interactions. As the “media equation” suggests, people are able to see objects (including robots) as social agents and not just as a tool (Reeves and Nass, 1996). The present research uses this observation to apply theory and methods from the psychology of intergroup relations (normally used to investigate prejudice towards minorities or different ethnic groups, Pettigrew and Tropp, 2008) to understand how contact with non-human agents affects people's beliefs. Taken together, viewing social robots as similar to a minority (human) group, opens the possibility for scientists and practitioners to apply and benefit from a rich history of research on intergroup relations; along with suggesting practical interventions to facilitate attitude change and engender more realistic expectations about technology.

### 5.2 Limitations and future directions

One limitation of the present studies is that attitudes were measured immediately following the contact procedures. Therefore, we do not know to what extent the changes we induced last over time or influence behaviour. Similar interventions have been shown to promote relatively enduring changes in participants' attitudes towards groups of humans (Eller and Abrams, 2004), as well as positive expectations about interactions and responses during actual interactions (Mallett and Wilson, 2010; West and Turner, 2014). For example, West and Turner (2014) found that participants who watched a video of a positive interaction between two strangers, one of whom they were led to believe had schizophrenia, displayed more positive non-verbal behaviors in a subsequent interaction with someone who they believed had schizophrenia, compared to participants who watched the same video without being told that the person had schizophrenia. This evidence suggests that direct and indirect contact with social robots may produce lasting and meaningful changes in attitudes, however, longitudinal studies are needed to explore this.

Finally, it is worth noting that the vast majority of participants in the present research had little or no experience with humanoid robots. Therefore, it was not possible to test whether the effect of contact on attitudes differed as a function of participants' prior experience. Future research could usefully seek to recruit participants with a range of experiences in order to test whether and how the effects of different contact procedures are moderated by prior experience, much in the same way as prior experience has been shown to moderate the effect of contact on people's attitudes towards other people (Dhont and Van Hiel, 2011).

## Declarations

### Author contribution statement

Marina Sarda Gou: Conceived and designed the experiments; Performed the experiments; Analyzed and interpreted the data; Wrote the paper.

Thomas L. Webb, Tony J. Prescott: Conceived and designed the experiments; Analyzed and interpreted the data; Wrote the paper.

### Funding statement

This work was supported by 10.13039/100010269Wellcome Trust (214963/A/18/Z) and 10.13039/100010661Horizon 2020 Framework Programme (Human Brain Project SGA-2, 785907; Human Brain Project SGA-3, 945539).

### Data availability statement

Data associated with this study has been deposited at osf.io/a9tqb/.

### Competing interest statement

The authors declare the following conflict of interests: TJP is a director and shareholder of the company Consequential Robotics Ltd that develops social robots, and of the company Cyberselves Ltd that develops middleware for robotic systems. MSG and TLW have no competing interests.

### Additional information

No additional information is available for this paper.
